# Clinical Decision Support systems: A step forward in establishing the clinical laboratory as a decision maker hubA CDS system protocol implementation in the clinical laboratory.

**DOI:** 10.1016/j.csbj.2023.08.006

**Published:** 2023-08-19

**Authors:** Emilio Flores, José María Salinas, Álvaro Blasco, Maite López-Garrigós, Ruth Torreblanca, Rosa Carbonell, Laura Martínez-Racaj, Maria Salinas

**Affiliations:** a Clinical Laboratory, University Hospital Sant Joan d'Alacant, Crta. Nacional 332 s/n, 03550, San Juan de Alicante, Spain; b Department of Clinical Medicine, Universidad Miguel Hernández, Crta. Nacional N-332 s/n, 03550, San Juan de Alicante, Spain; c Informatics Technology and Communication Department, University Hospital Sant Joan d'Alacant, Crta. Nacional 332 s/n, 03550 San Juan de Alicante, Spain; d Fundación para el Fomento de la Investigación Sanitaria y Biomédica de la Comunitat Valenciana (FISABIO), Av. de Cataluña 21, 46020, Valencia, Spain

**Keywords:** Clinical Decision Support Systems, Clinical laboratory, Electronic health records, Quality improvement, Operational Management, Artificial intelligence

## Abstract

**Background:**

New tools for health information technology have been developed in recent times, such as Clinical Decision Support (CDS) systems, which are any digital solutions designed to help healthcare professionals when making clinical decisions. The study aimed to show how we have adopted a CDS system in the San Juan de Alicante Clinical Laboratory and facilitate the implementation of our protocol in other clinical laboratories. We have user experience and the motivation to improve healthcare tools. The improvement, measurement, and monitoring of interventions and laboratory tests has been our motto for years.

**Materials and methods:**

A descriptive research was conducted. All stages in the design of the project are as follows: 1. Set up a multidisciplinary workgroup. 2. Review patients’ data. 3. Identify relevant data from main sources. 4. Design the likely outcomes. 5. Define a complete integration scenario. 6. Monitor and track the impact. To set up this protocol, two new software systems were implemented in our laboratory: AlinIQ CDS v8.2 as Rule Engine, and AlinIQ AIP Integrated Platform v1.6 as Business Intelligence (BI) tool.

**Results:**

Our protocol shows the workflow and actions that can be done with a CDS system and also how it could be integrated with other monitoring systems, as well as some examples of KPIs and their outcomes.

**Conclusions:**

CDS could be a great strategic asset for clinical laboratories to improve the integration of care, optimize the use of laboratory tests, and add more clinical value to physicians in the interpretation of results.

## 1 Background

There is an increasing focus on health information technology (HIT) and how data from different healthcare settings are been digitalized [1]. This has been a significant and positive development for Laboratory Medicine (LM), with end-to-end barcode-based tracking of samples, advanced Laboratory Information System (LIS), improved middleware, and analytics [2].

Over the past decades, laboratory specialists have focused mainly on the analytical step of the laboratory investigations process. LM today is highly automated and provides high-quality tests results for numerous different parameters from several different specimens in a short time. This technological function is usually well-managed by laboratory technicians. However, as described by *Lundberg, G.D.* [3], the processes with the highest number of errors are those that have lesser control by laboratory specialists, namely, [3] test requests and actions to be taken after test results are received. This can be corrected through Demand Management (DM) interventions, and Result Management (RM) interventions [4].

In recent times, HIT advances have revolutionized the laboratory medicine model. There has been a progression from a Traditional Laboratory, which passively measured the ordered tests and just corroborated or ruled out the clinician’s suspected hypothesis, to a Technological Laboratory, focused on technology to deal with test demand overload, and that measures its success through intermediate indicators, such as the number of measured tests and costs. Finally, the model evolution is reaching a “Leading Laboratory” [5]. A model that does not just intervene in clinical decision-making but also makes clinical decisions, and measures its success and shortages through performance indicators, e.g. “new diagnosed cases”. Thus, it could be argued that the Leading Laboratory model is more patient-focused than ever before, as its main goal is to identify undiagnosed disorders that would otherwise have remained hidden. There is a lot of evidence supporting this fact, since it is believed that diseases such as diabetes [6], primary hyperparathyroidism [7], pancreatitis [8] or chronic kidney disease [9] are underdiagnosed (or difficult to diagnose) among the population. Today, LM is contributing to most medical decision-making through knowledge, communication, leadership, HIT use, partnership with clinicians, and creative imagination [10].

Lately, several new tools have been developed for HIT, e.g., Clinical Decision Support (CDS) systems, which are defined as any digital solution designed to help healthcare professionals make clinical decisions [11]. CDS has been useful for several healthcare-related functions and settings, such as pharmacy, emergency department (ED), and intensive care units [12], [13]. However, within laboratories, their major use has been limited to Computerized Provider Order Entry (CPOE) systems [14], [15]. CDS systems encompass a rich set of tools that have the potential to drive significant improvements in laboratory testing; indeed, apart from diagnostic support, laboratories have a lot of reasons to implement them: cost containment, patient safety through the reduction of prescription errors, clinical management through easier adherence to clinical guidelines, and workflow improvements [13]. Moreover, other tools related to Artificial Intelligence (AI), including machine learning, have been used in various fields like LM with the same purposes. However, the main challenge is still their application in daily clinical practice [16], [17].

In San Juan de Alicante Clinical Laboratory, we have been working for years to give appropriate use to all diagnostic and therapeutic tools that directly or indirectly are under our responsibility. This fact has been our motivation, and every displayed intervention has been thoroughly evaluated and monitored through appropriateness indicators, even reaching the national level [18]. Nowadays, leadership and digital solutions are our best allies and lead us to believe that LM has gone from “just testing” to “saving lives” [19].

This study aimed to show how we have adopted a CDS system in our clinical laboratory and to facilitate the implementation of our protocol in other clinical laboratories where there is a need or desire to integrate, automate and monitor RM, internal processes, and DM interventions.

## 2 Materials and methods

A descriptive research has been conducted to implement this protocol. A hypothesis is not being tested here, rather, the present study explores and details work practices to inform about the implementation of a CDS tool. The research is drawn on the elements and attributes of previous design methodologies reported in the literature.

### 2.1 Laboratory and hospital characteristics

Our clinical laboratory is located in a 396-bedded suburban University Community Hospital (Hospital Universitario San Juan de Alicante-Sant Joan d′Alacant) that serves people from the Department of Health (DH) “Alicante-Sant Joan d′Alacant”. 238.521 inhabitants (report from the year 2022) constitute this DH, distributed among the central hospital and nine Primary Care (PC) centers. The DH is integrated into the public health network of the Valencian Community, Spain.

Our laboratory receives samples from inpatients, outpatients, and PC patients collected at PC centers. The samples are dispatched through the mail and delivered to the laboratory sample reception desk.

### 2.2 Stages in the design

During the implementation of our CDS solution tool, we defined the various steps below:

1. Setting up a multidisciplinary workgroup: the interdisciplinary team included a group of subject matter experts, including workflow specialists from the clinical laboratory, information technology experts from the DH, CDS consultants, data managers from Business Intelligence (BI), and a project lead.
2. Reviewing patient data: the regional healthcare information system (SIP) captures information about inhabitants who have health insurance cards. There are two Electronic Medical Records (EMRs), namely, the PC system (*Abucasis –* Conselleria de Sanitat Universal i Salut Pública, Generalitat Valenciana, Spain) and the hospital system (*ORION CLINIC v.13 –* Conselleria de Sanitat Universal i Salut Pública, Generalitat Valenciana, Spain). Each EMR is accessed by physicians based on their profile as providers of primary or secondary care. Laboratory tests are requested electronically through the CPOE system from the EMR. The requests are recorded automatically in LIS, and the reports are automatically sent to the EMR. For inpatients, codes for diagnoses and clinical procedures are assigned by the International Classification of Diseases list codes (ICD-10) [20] on discharge.
3. Identifying relevant data from main sources: Fig. 1 shows data sources and information retrieved. From LIS, it is mandatory to receive every request as well as the complete 5-year historical results for every patient in the CDS. From CPOE, every new request needs to be transmitted to the CDS. From the EMR, heterogeneous data, including diagnosis, demographic information, vital signs, electronic request (ER) triage, pharmacological treatment, nurses’ scores, clinical notes, and anthropometric data are retrieved. From the primary care EMR, the CDS retrieves any active or historical diagnoses classified according to ICD codes. From SIP, data about insurance, PC, and doctor assigned are obtained. From the coding system (*IRIS* software – Conselleria de Sanitat Universal i Salut Pública, Generalitat Valenciana, Spain), audited data of the diagnosis based on ICD-10 classification [20] and clinical procedures are obtained.

**Fig. 1:**
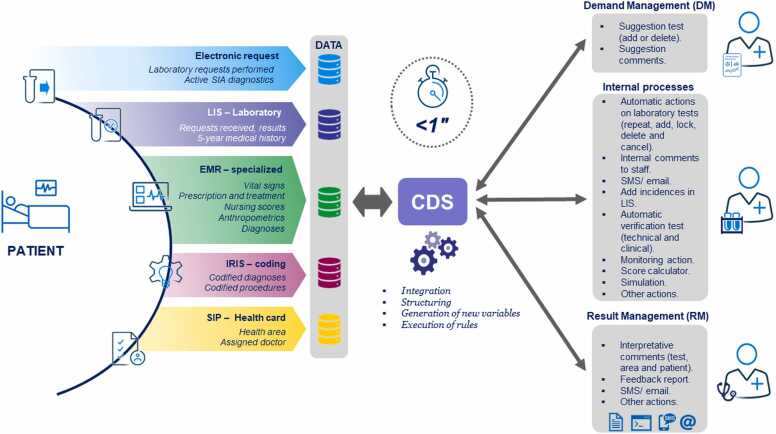
The figure shows the action workflow of the Clinical Decision Support (CDS) system. First, data sources and information retrieved from several databases are shown. Second, data management key processes are displayed. Third, some CDS actions’ examples at different levels are given.
4. Predicting possible outcomes indicated by the CDS system based on laboratory interests, bibliography search, and technical and integration capabilities of the infrastructure.
5. Defining a complete integration scenario that allows interoperability between all systems involved in the workflow and creates structured mechanisms to transmit patient data.
6. Monitoring and tracking the impact of the system through a BI software like AlinIQ Integrated Platform.

### 2.3 Tools acquired

Two new software were deployed in our laboratory: AlinIQ CDS v8.2 as Rule Engine (Abbott Laboratories, Chicago, ILL, US), to manage pathways and actions, as well as a BI holistic solution for key indicators monitoring and measurement: AlinIQ Integrated Platform (AIP) v1.6 (Abbott Laboratories, Chicago, ILL, US).

### 2.4 Communication

Each workgroup defines the process for internal communication within it. Members of each group are provided with feedback related to their function, and a leader is designated for every group. These leaders, together with the laboratory manager, are responsible for communicating with other clinical teams regarding strategies and pathways.

## 3 Results

### 3.1 Workflow

AlinIQ CDS system is a back-end rule engine that applies logic to predict outcomes whenever a trigger event occurs in the laboratory workflow. Events selected to activate the CDS are order requests, sample arrivals at the laboratory, and actions delivered for a concrete test in the LIS.

Whenever any of these triggering events occurs, the integration hub collects, transforms, and sends the data to the decision-making engine. All data are appropriately structured and chronologically sorted.

All communication and performance tests of the system were performed to ensure appropriate response time, the integrity of data, and no performance-related issues.

### 3.2 Actions

Fig. 1 shows the actions committed at different levels - at DM level (overuse and underuse), at RM level (laboratory report), and at the internal processes level (sending data to BI). In order to exemplify one of the multiple actions that can be done, it is worth mentioning one applied at the DM/internal processes level related to the prediction of urinary tract infection (UTI) diagnosis in the ED, following the examples available in the literature [21]. Communication with predictive algorithms was deployed in the integration engine. The algorithms require a set of laboratory results per patient to give a prediction of UTI risk. Whenever this data is available, it is sent to the algorithms script to receive the prediction through CDS. Subsequently, the rules implicated are applied by the CDS engine to obtain clinically relevant actions in the context of UTI management.

### 3.3 Monitoring system

Any outcome produced by the CDS system is reported to the BI software as a transformed Key Performance Indicator (KPI). Through BI, the laboratory can monitor the impact of all rules and interventions automated with the CDS system and get personalized metrics. Following our laboratory mission, a set of custom reports are periodically designed [5] which are now also integrated with the BI to understand operational performance at three intervention levels: DM, internal processes, and RM. Table 1 shows some examples of KPIs and their outcomes:

**Table 1:** Examples of Key Performance Indicators (KPIs) at the three laboratory intervention levels: demand management (DM), internal processes, and result management (RM). Outcomes and study periods from different interventions are displayed, as well as CDS intervention details of interest. *Results published in: [21].* *Calculated reagent costs.

Examples of Key Performance Indicators (KPIs)				
Intervention level	KPIs	Outcomes	Study period	Intervention details
**Demand management (DM)**	Number of new hypercalcemia of malignancy cases in the ED	186 cases	January 2022 - June 2023	CDS registers serum total calcium in every ED patient request aged 40–90 with a known malignant disease
	Number of removed thyroid test over requests	3 670 tests (Savings of 5 395 Euros) * *		CDS removes a thyroid test when requested in the previous 3-months and unknown thyroid disease
**Internal processes**	Post-analytical turnaround time (TAT)	27.5 min vs. 2.5 min	June 2021 vs. June 2023	Time spent from test result retrieval in the laboratory to its availability in the ED
	Percentage of auto-verification	75% vs. 91%		Calculation of the percentage of tests automatically verified with respect to the total number of tests performed
**Result management (RM)**	Number of primary care (PC) patients classified with low risk of heart failure (HF) after NT-proBNP interpretation	370 patients	June 2022 - June 2023	CDS registers an interpretative comment (according to clinical guidelines recommendations) regarding patient HF risk in patients with unknown HF.
	Number of cases with pancreatitis prognosis in the ED	180 patients		CDS calculates the Ranson and SIRS scores for the LIS to report a prognostic comment on any ED patient with high pancreatic enzymes values.

Monitoring through intelligent solutions such as AIP is the way to detect and consequently minimize errors, reduce unnecessary test requests, and allow physicians to spend their time on other tasks. Regarding intervention examples provided in Table 1, we can see how KPIs have a direct impact on patient support:

- The first example is related to those cases where serum total calcium test is under-requested in the ED: KPIs allow us to know how many calcium determinations have been added thanks to a DM intervention integrated with the CDS system and consequently, the number of new patients diagnosed with hypercalcemia of malignancy [22]. The next DM example is about thyroid test over-request; KPIs monitor how many thyroid test requests have been removed every time a patient had a previous result (previous 3 months) and unknown thyroid disease.
- Regarding internal processes, KPIs measure post-analytical turnaround times (TATs) between two periods to know how CDS interventions are acting compared with pre-intervention times: outcomes show reduced ED TATs, as well as an increase in the percentage of tests auto-verified.
- Finally, at the RM level, the interpretation of NT-proBNP values in PC patients is key: KPIs monitor the number of test requests from PC and the number of patients classified according to the risk of heart failure (HF). Pancreatitis is also monitored, and KPIs show the number of ED patients with reported pancreatitis prognosis after a CDS intervention.

### 3.4 Non-real-time project

Another use of the CDS tool is a non-real-time project which simulates the target protocols and current clinical guidelines. It allows the evaluation of the potential impact of any clinical pathway and even the redesign of the interventions and KPIs before implementation.

## 4 Discussion

Our study shows the adoption of a CDS system in a clinical laboratory of a DH in the Valencian Community, Spain. We have found little literature on research about CDS in LM: only one recent study made a retrospective evaluation of the tool [23]. That is why we think our experience, interventions and outcomes could be very interesting and transferable to other laboratories.

Identifying risks and barriers prior to the adoption of a new system is very important for the success of the project. In our experience, the key factors were training of the workgroup, internal communication between them, and strong leadership. Moreover, it is crucial that laboratory managers encourage staff to identify and bring forward all functionality-related issues [24], [25].

Effective deployment of the CDS requires thoughtful design and careful maintenance [2]. When designed carefully, it empowers clinicians to use laboratory tests more effectively and deliver higher-quality patient care. This solution aims to provide clinical support in areas where we anticipate a high number of errors*. Stroobants AK* et al. [4] showed a 12% error in DM (pre-pre-analytical stage) and a 5% error in RM (post-post-analytical stage). The design of these tools is conditioned to the areas where the laboratory wants to perform an action [26], [27]. Finally, it is important to spend time on identifying key data, designing the data model, and the throughputs desired [25].

AI can rapidly and accurately sift through large data and uncover correlations and patterns that are imperceptible to human cognition [28]. Hence, it presents a significant opportunity for the improvement of prevention, diagnosis, monitoring, and treatment in different healthcare settings [29], [30]. However, including it in the clinical workflow is challenging, so one of the feasible alternatives is the design and deployment of a CDS system having these capabilities.

The exponential growth of clinical knowledge and availability of massive clinical data is a challenge for the clinician with limited time; however, it is also an opportunity [5]. There has never been a greater need for the laboratory to contribute to medical decision-making; nevertheless, there is a gap between daily practice and the potential of new tools [5]. There has been a reassessment about the role of the laboratory specialist within the healthcare system and new developments in HIT, such as the integration of data from different sources like EMR or CPOE. LIS can increase efficiency, productivity, and agility in the clinical workflow [9], [22]. Therefore, there is an opportunity to refocus on the role of a laboratory professional [22], [31].

It should be noted that the current study has some limitations. Regarding the results provided, we are not showing quantitative outputs as it is out of the scope of this descriptive research. Outcomes of interventions implying CDS and AIP tools have been reported in Table 1 to provide the reader with real examples. Further studies (whether with positive or negative outcomes) will also be reported in the literature as soon as possible. An additional limitation worth mentioning is the difficulty of its application within a non-digitalized environment, as the protocol needs access and inputs from several databases to provide the CDS system with quality information.

## 5 Conclusions

In conclusion, disruptive technology is an opportunity to include this new laboratory model, provided with digital health solutions, in the clinician’s workflow and lead the diagnostic process. Moreover, it could be a significant strategic asset for clinical laboratories to improve the integration of care, optimize the use of laboratory tests through DM, and add more clinical value to physicians in the interpretation of results via RM.

## Ethical approval

Not applicable.

## Source of Funding

None declared.

## Credit authorship contribution statement

Conceptualization: Emilio Flores, María Salinas, José María Salinas, Data curation: not applicable, Formal analysis: Emilio Flores, María Salinas, José María Salinas, Funding acquisition: not applicable, Investigation: Emilio Flores, María Salinas, Laura Martínez-Racaj, Methodology: Emilio Flores, María Salinas, Álvaro Blasco, Maite López-Garrigós, Ruth Torreblanca, Rosa Carbonell, Laura Martínez-Racaj, Project administration: Emilio Flores, María Salinas, Álvaro Blasco, Maite López-Garrigós, Ruth Torreblanca, Rosa Carbonell, Laura Martínez-Racaj, Resources: Emilio Flores, María Salinas, Software: José María Salinas, Supervision: Emilio Flores, María Salinas, Validation: Emilio Flores, María Salinas, Álvaro Blasco, Maite López-Garrigós, Ruth Torreblanca, Rosa Carbonell, Visualization: Emilio Flores, María Salinas, Roles/Writing - original draft: Emilio Flores, Laura Martínez-Racaj, Writing - review & editing: Emilio Flores, Laura Martínez-Racaj,

## Declaration of Competing Interest

None declared.
